# *CmFTL2* is involved in the photoperiod- and sucrose-mediated control of flowering time in chrysanthemum

**DOI:** 10.1038/hortres.2017.1

**Published:** 2017-02-15

**Authors:** Jing Sun, Heng Wang, Liping Ren, Sumei Chen, Fadi Chen, Jiafu Jiang

**Affiliations:** 1 College of Horticulture, Nanjing Agricultural University, Nanjing 210095, China

## Abstract

The chrysanthemum genome harbors three *FT-like* genes: *CmFTL1* and *CmFTL3* are thought to act as regulators of floral induction under long-day (LD) and short-day (SD) conditions, respectively, whereas the function of *CmFTL2* is currently unclear. The objective of the present research was to explore the function of *CmFTL2* in the determination of flowering time of the photo-insensitive chrysanthemum cultivar ‘Floral Yuuka’, both in response to variation in the photoperiod and to the exogenous provision of sucrose. Spraying leaves of ‘Floral Yuuka’ plants with 50 mM sucrose accelerated flowering and increased the level of *CmFTL2* transcription in the leaf more strongly than either *CmFTL1* or *FTL3* under both long and SD conditions. Transcription profiling indicated that all three *CmFTL* genes were upregulated during floral induction. The relationship of the *CmFTL2* sequence with that of other members of the *PEBP* family suggested that its product contributes to the florigen rather than to the anti-florigen complex. The heterologous expression of *CmFTL2* in the *Arabidopsis thaliana ft-10* mutant rescued the mutant phenotype, showing that *CmFTL2* could compensate for the absence of *FT*. These results suggest that *CmFTL2* acts as a regulator of floral transition and responds to both the photoperiod and sucrose.

## Introduction

The switch from vegetative to reproductive growth is a key event in the life cycle of plants. This switch is triggered by a variety of both environmental cues (notably photoperiod and temperature) and endogenous signals (hormonal status and carbohydrate availability) sensed by the shoot apical meristem.^1–4^ A change in the photoperiod stimulates a network of regulatory genes, one of the most important of which is *FT*. *FT* integrates signals from various relevant pathways^5^ and is transported via the phloem to the shoot apex.^6–9^ The protein encoded by *FT* belongs to a small family whose members share homology with the mammalian phosphatidyl ethanolamine-binding proteins (PEBPs). The genome of the model angiosperm *Arabidopsis thaliana* harbors, in addition to *FT*, the related genes *TSF*, *BFT*, *ATC*, *MFT* and *TFL1*, all of which are involved in floral induction.^5,10–13^ *FT* or *FT-like* genes have been described in a growing number of plant species, including the chrysanthemum.^14–22^

Sugars not only represent the primary source of carbon and energy but also act as regulator molecules controlling metabolism, the stress response, growth and development. Sucrose, the predominant form of sugar in plant tissue, has been shown to promote flowering in various plant species.^3,21,22^ Shortly before floral initiation in *A. thaliana* plants exposed to SD conditions, the level of both sucrose and gibberellins (GA_4_) in the shoot apex increases markedly.^23^ In *Sinapis alba*, phloem sap sucrose levels rise during floral induction under both displaced SD and LD conditions.^24^ In *Xanthium strumarium*, exposing a leaf to a single inductive photoperiod rapidly increases its sucrose content.^25^ Nevertheless, there is no evidence for the accumulation of sucrose during floral transition, suggesting that its role in the flowering process is to contribute to sucrose homeostasis in a species-specific manner.^3^ A genetic analysis has shown that in *A. thaliana,* loci associated with the determination of flowering time map to the same genomic region as those determining the content of certain carbohydrates, a coincidence that has been taken to imply a functional relationship between carbohydrate levels and flowering time.^26^ Sugars present in the leaf can act as a mobile signal to raise the level of SQUAMOSA PROMOTER BINDING PROTEIN-LIKE, thereby triggering floral transition in *A. thaliana*.^27^

Chrysanthemum is a popular ornamental species, second only to the rose.^28^ The summer-to-autumn flowering cultivar ‘Floral Yuuka’ flowers under both LDs and SDs, although it flowers earlier under SD conditions. RNA-seq analysis has suggested the involvement of the sugar signaling pathway in its floral transition under LD conditions.^29^ *CsFTL3* is a key regulator of photoperiod-induced flowering under short days in chrysanthemum,^30^ whereas *CsFTL1* is probably required for flowering under LDs.^31^ The function of *CsFTL2* has not yet been defined. Here, the *CmFTL* copies present in ‘Floral Yuuka’ were characterized. In particular, transcriptional profiling was used to show that the *CmFTL2* transcript accumulates during floral transition in plants exposed to both SDs and LDs. The abundance of the transcript is increased when the plants are provided with exogenous sucrose, a treatment that also accelerates flowering. The heterologous expression of *CmFTL2* in *A. thaliana* rescues the phenotype of an *FT* loss-of-function mutant. Our results suggest that the involvement of *CmFTL2* in floral induction operates through one or more photoperiod- and sucrose-regulated pathways.

## Materials and methods

### Plant materials and growing conditions

Chrysanthemum (cultivar ‘Floral Yuuka’) plants were maintained by the Chrysanthemum Germplasm Resource Preserving Centre (Nanjing Agricultural University, China). Cuttings of uniform height (6–7 cm) and vigor were selected for the present experiments and were rooted and grown in a greenhouse under a 16 h photoperiod provided by 300 μE m^−2^ s^−1^ lighting. The lit period temperature was kept in the range of 21–25 °C, and the dark period temperature was maintained at 15–17 °C.

### The isolation of *CmFTL1*, *CmFTL2* and *CmFTL3* cDNAs and a phylogenetic analysis of their sequences

Total RNA was extracted from snap-frozen leaves using a Plant RNA Extraction Kit (Takara Bio, Dalian, China), following the manufacturer’s protocol. A 1 μg aliquot was reverse-transcribed in a 10 μL reaction volume based on the Superscript First-Strand Synthesis System (Invitrogen, Carlsbad, CA, USA). For the subsequent amplification of the *CmFTL* open reading frame (ORF) sequences, a 1 μL aliquot of the cDNA preparation (equivalent to 100 ng) was used as the template in reactions primed by oligomers targeting *CmFTL1*, *CmFTL2* or *CmFTL3*.^30^ The PCR protocol comprised a 94 °C/3 min denaturation, followed by 32 cycles of 94 °C/30 s, 55 °C/30 s and 72 °C/60 s and was completed by a 72 °C/7 min extension. The amplicons were introduced separately into the pMD19-T Simple Vector (Takara Bio) for sequencing. The remainder of each cDNA sequence was acquired using RACE-PCR following methods described elsewhere.^32^ The deduced polypeptide sequences were subjected to a BLASTp search (www.ncbi.nlm.nih.gov/blast/) to identify homologs, and the members of the PEBP family were clustered using Clustal X software (www.clustal.org/clustal2/). A neighbor-joining tree was generated using MEGA 5 software (www.megasoftware.net), applying the Poisson model with gamma-distributed rates and 1000 bootstrap replicates.

### Semi-quantitative RT-PCR and real-time quantitative RT-PCR

A 1 μg aliquot of total leaf RNA, extracted as described above, was reverse-transcribed in a 10 μL reaction volume using the Superscript First-Strand Synthesis System (Invitrogen). Real-time quantitative RT-PCR (qRT-PCR) analyses were conducted in accordance with MIQE guidelines.^33^ The primers targeting the three *CmFTL* genes (sequences presented in Supplementary Table S1) were designed based on the sequence close to the 3′ end of each gene, including the 3′-UTR.^30^ All amplicons were sequenced to confirm their identities. The qRT-PCRs were performed using a SYBR Premix Ex Taq Kit (Takara Bio), in accordance with the manufacturer’s protocol. The PCR protocol comprised a 95 °C/2 min denaturation, followed by 40 cycles of 95 °C/15 s, 55 °C/15 s, and 68 °C/20 s. The products were subjected to a melting curve analysis. The *EF1α* gene^30^ was used as the reference sequence.

### Temporal profiling of *CmFTL* transcription

A set of 300 rooted ‘Floral Yuuka’ cuttings of uniform length was grown in a greenhouse maintained at a temperature of ~22 °C under an 8 h photoperiod (hereafter, SD), including a 4 h night break (NB) (between 22:00 and 02:00) provided by high-pressure sodium lumps. The SD+NB regime was maintained until the floral bud was visible in the plants. The third leaf below the apex of three plants was harvested at 16:00 every other day between days 67 and 104 (flowering stage) based on a previously described method.^34^ A second set of 300 cuttings was exposed to SD+NB up to day 73, then to SD; leaves were sampled from these plants every other day at 16:00 between days 73 and 88. RNA was extracted from snap-frozen leaf samples, as described above. The experiment was replicated at least twice. Shoot apices sampled at various developmental stages were fixed in formalin: acetic acid: alcohol (v/v, 5:5:90), embedded in Paraplast (Sigma, Shanghai, China), cut into 10 μm sections and stained with 1% w/v toluidine blue.

### The induction of flowering by sucrose treatment

A set of 300 ‘Floral Yuuka’ cuttings was grown under an SD+NB regime up to the 15 leaf stage, at which point three equal groups of 100 plants each were formed. One group received an application of aqueous 50 mM sucrose sprayed onto their leaves every four days; a second group was simultaneously sprayed with water; plants’ leaves in the third group was semi-defoliated. We sectioned the stem tip by hand and examined it using a stereomicroscope. We used the time when the shoot apical meristems begin to expand as the indicator of floral bud initiation.

### The transcriptional response to sucrose treatment

One hundred of ‘Floral Yuuka’ rooted cuttings of height 10 cm were grown under a SD+NB (light period 23 °C, dark period 20 °C) regime for 20 days, by which time they had developed ~10 leaves. Fifty plants were switched to a SD (8 h photoperiod, lit period 23 °C, dark period 20 °C) regime. After three additional days, the transcriptional response of the *CmFTLs* to sucrose treatment was examined by first withholding water for seven days and then spraying to dripping with aqueous 50 mM sucrose; a control set of plants was sprayed with water. The third leaf below the apex was harvested at various time points and analyzed by qRT-PCRs targeting the *CmFTLs*, following the procedures described above for RNA extraction and reverse transcription.

### Functional complementation of the *A. thaliana ft-10* mutation by *CmFTL2*

The 6977 bp *A. thaliana FT* promoter from the pENTR4-*AtFT* promoter vector was introduced into the GATEWAY vector pHGW using LR Clonase II enzyme mix (Invitrogen) to produce the construct pHGW-*proFT*.^35^ The full-length *CmFTL2* coding sequence was introduced into the recombined pHGW-*proFT* plasmid at its *Xho*I and *Pst*I cloning sites to produce the construct *proFT::CmFTL2*. The *A. thaliana ft-10* mutant^36^ was transformed using the floral dip method.^37^ Two independent transgenic lines were selected by regenerating plants on a Murashige and Skoog (1962) medium containing 25 mg L^−1^ hygromycin. The presence of the transgene was confirmed by an RT-PCR analysis of RNA extracted from leaves, using primers directed to the *CmFTL2* ORF (Supplementary Table S1). *A. thaliana ACT2* (*At3g18780*) was used as the reference sequence. The phenotypic effect of the transgene was recorded in the T_1_ generation. Significance testing was performed using SPSS 20.0 (http://www-01.ibm.com/software/cn/analytics/spss/).

## Results

### Sucrose promoted early flowering in ‘Floral Yuuka’

**Figure 1:**
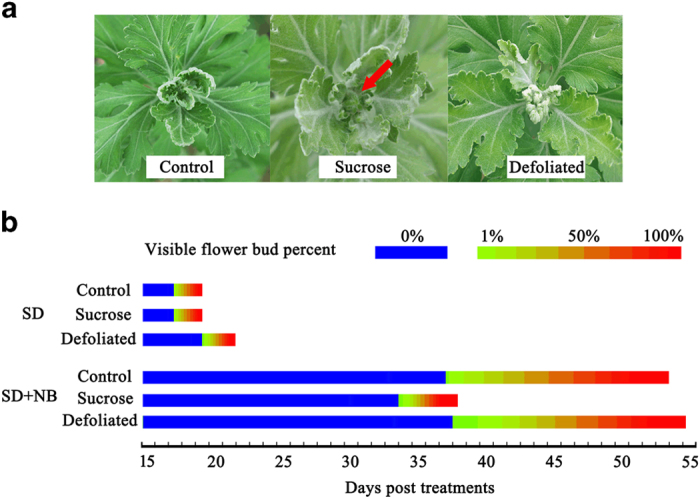
The effect of spraying the leaves of ‘Floral Yuuka’ plants with a sucrose solution. (**a**) Floral initiation in plants grown under an SD+NB regime. Flower buds are arrowed. (**b**) The proportion of plants forming flower buds in response to various treatments (*n*=100).

The effect of sucrose on floral transition in ‘Floral Yuuka’ was examined by growing plants under a SD plus night break (SD+NB) regime and then spraying them with a sucrose solution. Floral buds became visible on the sucrose-treated plants 34 days after the start of the treatment, and every plant had reached this stage by 38 days (Figure 1b). By contrast, at that time, only 70% of the water-treated plants and 40% of the semi-defoliated plants showed visible buds. After an additional 14 days, the proportion of plants in either of these control treatments that had reached flowering was still not 100%. Under SD conditions, there was no discernible treatment effect, as all three sets of plants reached flowering at the same time (Figure 1b). Thus, the sucrose treatment clearly accelerated flowering in ‘Floral Yuuka’ plants grown under an SD+NB regime. In addition, we also performed the same sucrose treatment on ‘Jimba’, a short-day cultivar. We found no differences between the experimental group (sucrose-treated) and the control group (water-treated) in terms of the floral bud differentiation time, under both LD and SD conditions. Neither group bolted under the LD conditions (data not shown).

### The ‘Floral Yuuka’ *CmFTL* alleles

**Figure 2:**
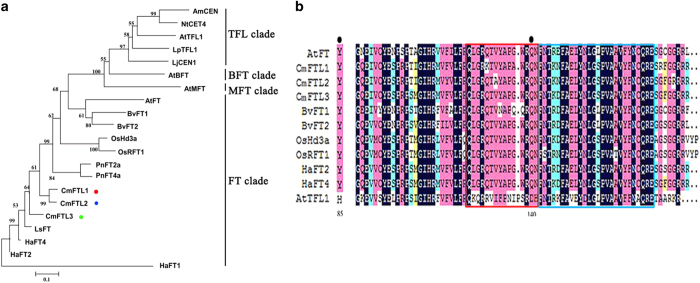
*CmFTL* polypeptide sequences. (**a**) Phylogeny of the *PEBP* family: *A. thaliana AtFT* (AT1G65480), *AtBFT* (AT5G62040), *AtMFT* (AT1G18100), *AtTFL1* (NP_196004), *Antirrhinum majus AmCEN* (Q41261.1), *Populus nigra PnFT2a* (AB109804.1), *PnFT4a* (AB369074.1), *Lactuca sativa LsFT* (BAK14368.1), *Chrysanthemum seticuspe CsFTL1* (AB679270), *CsFTL2* (AB679271), *CsFTL3* (AB679272), rice *OsHd3a* (JX472280), *OsRFT1* (AB426873.1), tobacco *NtCET4* (AF145261), *Lotus japonicus LpTFL1* (AF316419), *LjCEN1* (AY423715), *Helianthus annuus HaFT2* (GQ884987.1), *HaFT4* (GU985573.1), *Beta vulgaris BvFT1* (HM448910.1), and *BvFT2* (HM448912.1). (**b**) Partial alignment of *PEBP* polypeptides. Filled circles indicate residues present in *FT* polypeptides but not in *FT-like*. Y85 and Q140 are essential for the formation of an external loop. The red and blue boxes highlight the conserved segments (B and C), required for *FT-like* activity.

A BLASTp search of the NCBI database showed that the ‘Floral Yuuka’ *CmFTL1*, *CmFTL2* and *CmFTL3* alleles each encoded the same polypeptide as their equivalent in *Chrysanthemum seticuspe*.^30^ A cross-species phylogenetic analysis of *PEBP* family members indicated that all three genes belong to the clade that includes *FT* (Figure 2a). Each sequence encoded the residues Y85 and Q140, which are required for the formation of an external loop, and the conserved B and C segments (Figure 2b).

### Temporal profiling of *CmFTL2* transcription

**Figure 3:**
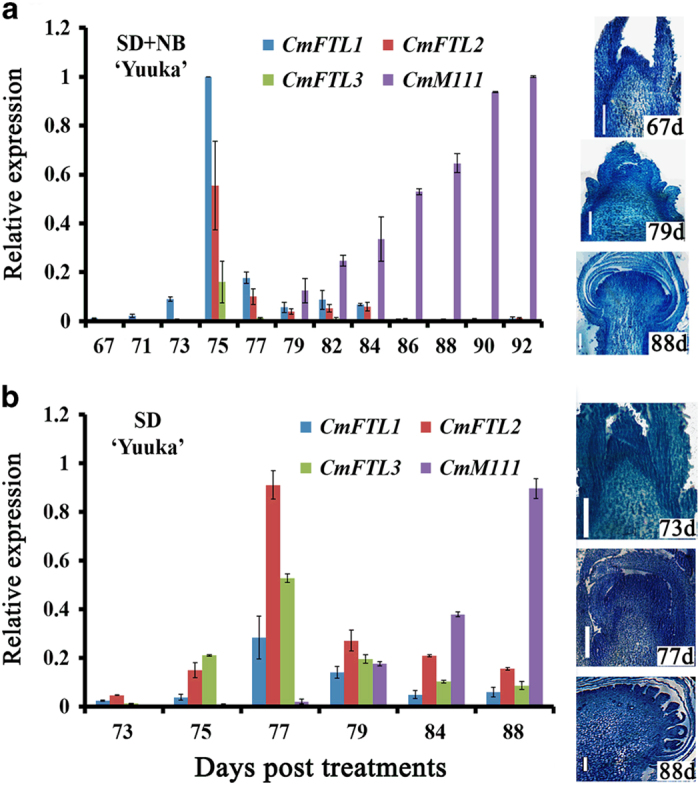
Temporal transcription of *CmFTLs* in ‘Floral Yuuka’, as assayed by qRT-PCR. Plants were grown under (**a**) an SD+NB or (**b**) an SD regime. Values reflect the mean of three biological replicates, with three technical replicates per biological replicate. The right panel illustrates shoot apices harvested at various developmental stages, embedded in Paraplast and stained with toluidine blue; bar: 1 mm.

When ‘Floral Yuuka’ plants were grown under the SD+NB regime for 116 days, the abundance of each of the three *CmFTL* transcripts increased between days 67 and 75 (Figure 3a). Following the appearance of the floral primordia, *CmM111* (which shares the same ORF as the *AP1* homolog *CsM111* (AY173054) was upregulated.^38^ The shoot apical leaf buds had reached the doming stage by day 79, and the shoot apical meristem's involucres had become fully formed by day 88. Plants that were no longer exposed to NB after day 73 (the SD treatment) reached the doming stage within an additional four days (day 77). Under these conditions, the flowering process was accelerated, with the first florets making their appearance on day 88 (Figure 3b). During the period of exposure to short days, *CmFTL2* was upregulated, whereas *CmFTL3* was upregulated to a greater extent than *CmFTL1*. Thus, *CmFTL2* transcription occurred during the period of floral transition under both SD+NB and SD conditions.

### Expression patterns of *CmFTLs*

**Figure 4:**
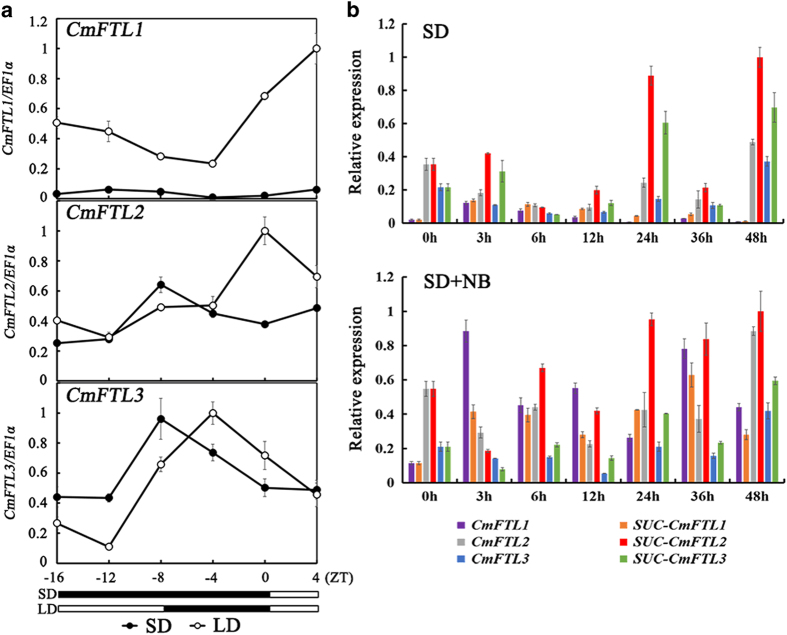
Diurnal variation in *CmFTL* transcription in ‘Floral Yuuka’ plants, as assayed by qRT-PCR. (**a**) Plants exposed to either an LD or an SD regime. The illumination regime is illustrated by white (lit) and black (dark) bars. (**b**) Plants sprayed with 50 mM sucrose and exposed to either an SD+NB or an SD regime. Values reflect the mean of three biological replicates.

To investigate the effect of light on *CmFTL* expression in ‘Floral Yuuka’, the expression patterns of the *CmFTLs* in leaves of ‘Floral Yuuka’ under different light conditions were examined (Figure 4a). All three *CmFTL* transcript levels were higher during the dark period compared with the light period. Compared with SD conditions, the transcript levels of *CmFTL1* were much higher under LD conditions. The *CmFTL2* transcript levels increased at the start of the dark period and peaked around the middle of the dark period under the SD conditions and at dawn under the LD conditions. The *CmFTL3* transcript levels peaked around the middle of the dark period under both SD and LD conditions.

### Sucrose treatment induced *CmFTL2* transcription

In plants exposed to either SD or SD+NB, *CmFTL2* transcription levels were strongly induced by sucrose (Figure 4b). *CmFTL2* and *CmFTL3* were both transcribed during the SD+NB treatment, with the former accumulating to a higher level than the latter, whereas the level of *CmFTL1* transcription declined during this period (Figure 4b). Thus, supplying sucrose exogenously had the effect of inducing *CmFTL2* and *CmFTL3* in the SD+NB treatment, but *CmFTL1* transcription was much more strongly influenced in the SD than in the SD+NB treatment.

### *CmFTL2* rescues the phenotype of the *A. thaliana ft-10* mutant

**Figure 5:**
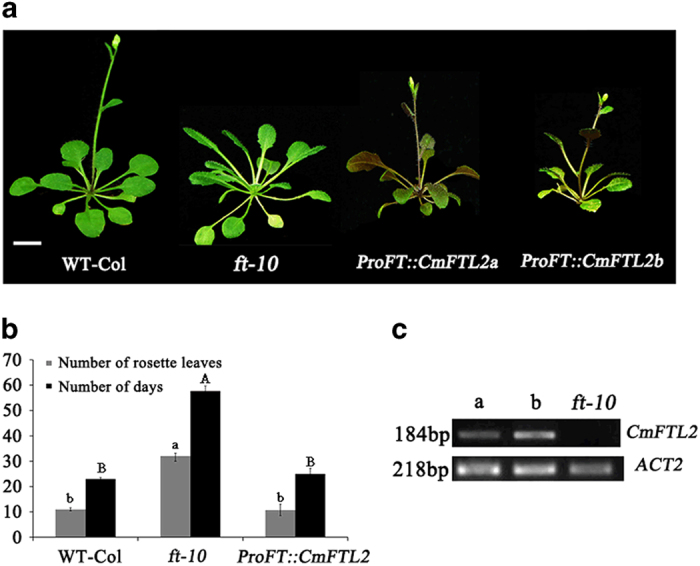
*CmFTL2* rescues the phenotype of the *A. thaliana ft-10* mutant. (**a**) Plants grown under an SD+NB regime. WT, wild type *A. thaliana*; *proFT::CmFTL2a* and *b*: two independent transgenic lines heterologously expressing *CmFTL2* in an *ft-10* mutant background; bar: 1 cm. (**b**) The number of rosette leaves and number of days required to reach flowering. Different letters show significant differences. Lowercase and uppercase letters represent the number of days and rosette leaves, respectively. Significance testing for both was performed at the 0.01 level. (**c**) Semi-quantitative RT-PCR analysis of *CmFTL2* transcription at the flowering stage. a and b: two independent transgenic lines heterologously expressing *CmFTL2* in an *ft-10* mutant background.

Flowering is delayed in the *A. thaliana ft-10* mutant.^39,40^ The native *AtFT* promoter was used to drive *CmFTL2* expression in a transformed *ft-10* mutant background to confirm its florigenic activity. In both independent transgenic lines, the flowering phenotype reverted to wild type, showing that *CmFTL2* was able to fully compensate for the absence of *FT* (Figure 5a). The number of rosette leaves formed per plant was 8–11 in the transgenic plants but 30–35 in the non-transgenic *ft-10* mutant (Figure 5b). A semi-quantitative RT-PCR assay applied to leaves harvested at the flowering stage confirmed the successful transcription of *CmFTL2* under LD conditions (Figure 5c).

## Discussion

At present, the uniformity of flowering time in summer-to-autumn type chrysanthemum is very important but difficult to control. Three *CsFTL* genes have been reported in chrysanthemum (a typical SD plant), and *CsFTL3* is a key regulator of photoperiod-induced flowering under short days in chrysanthemum.^30^ On the basis of over-expression experiments, the product of *CsFTL1* shows weak, florigenic activity. The gene is also induced by LD conditions. Hence, it has been proposed that *CsFTL1* encodes a LD florigen equivalent to *RFT1*.^31^ Here, the transcription of *CmFTL1* was higher than that of *CmFTL3* under the SD+NB condition in ‘Floral Yuuka’ (Figure 4b), which is consistent with *CsFTL1* in ‘Jimba’.^31^ We also wondered whether *CmFTL2* in SD and summer-to-autumn type chrysanthemums shows different photoperiod responses. The present comparison between the transcriptional behavior of ‘Floral Yuuka’ (summer-to-autumn type) raised under either an SD or an SD+NB regime showed that the abundance of the *CmFTL2* transcript increased in conjunction with floral initiation (Figure 3), suggesting that *CmFTL2* had a role during floral transition, which was inconsistent with the studies of SD chrysanthemum.^30^ Once the plants had experienced the SD+NB regime, the abundance of the *CmFTL1* transcript increased above that of *CmFTL3*. In addition, the gene was suppressed by the sucrose treatment. By contrast, the *CmFTL2* transcript continued to accumulate in sucrose-treated plants growing under either an SD or an SD+NB regime (Figure 4b), suggesting that in ‘Floral Yuuka’, both photoperiod and sucrose influence the transcription of the *CmFTLs*. Meanwhile, the expression of *CmFTL2* before and after the sucrose treatment in ‘Floral Yuuka’ could provide an improved understanding of the exquisite coordination that exists within the photoperiodic and sucrose-induced flowering gene network not only for chrysanthemum but also for various other plant species.

Sugar signaling is important for a wide range of developmental processes, including the regulation of floral induction.^27,41,42^ The most common sugar synthesized by plants is sucrose, which is a more stable molecule than either of its monosaccharide components glucose and fructose, making it a readily transportable compound. In photo-sensitive plants, the exposure of a leaf to a single inductive photoperiod results in a rapid increase in leaf sucrose content.^25^ Sucrose supply to the aerial portion of dark-grown plants does promote flowering in *A. thaliana*.^43^ In LD-grown *A. thaliana* plants, sucrose generated from photosynthetic activity induces the accumulation of the *SPL* transcript via the down-regulation of *miR156* ^27,42^ and amplifies the expression of *FT*.^44^ Because the transition from vegetative to reproductive development is tightly controlled, the flowering response of chrysanthemum cannot be fully explained by a photoperiod-based transcriptional regulation of florigen.^31^ We also performed a transcriptomic analysis. Our results suggested that while the photoperiod and the gibberellin pathways act in concert to promote the flowering of ‘Floral Yuuka’ plants grown under short days, sugar signaling is more important under LD conditions (data not shown).^29^ These data suggest that sucrose signaling may not be an important floral induction mechanism in ‘Floral Yuuka’ plants under short days.

In the last common ancestor of mono- and dicotyledonous species, *PEBP* family proteins include *FT-like* (encoding floral activators), *TFL1-like* (encoding floral inhibitors) and *MFT-like* (encoding factors involved in germination).^45^ The diversification of *TFL* from *FT* is thought to have occurred at least 150 mya (Figure 2a).^46^ The current evidence seems to indicate that the emergence of the *FT-like* genes coincided with the evolution of the flowering plants.^47^ In *A. thaliana*, the variable segment B and one ligand-binding pocket are both essential for *FT* function, whereas *FT-like* function requires segments B and C (Figure 2b).^48^ The segment B residues Q140 and D144 contribute to the function of both *FT* and *TF-like1*. Alignment of *FT* fourth exon sequences demonstrated that Q140 was present in *CmFTL2*, which suggests that *CmFTL2*, like *AtFT,* forms the external loop that provides an anion-binding site (Figure 2b). The implication is that the Y85 and Q140 residues allow *CmFTL2* to act as a florigen mediating the onset of flowering in chrysanthemum. The likelihood is that *CmFTL2* contributes to the florigen rather than the anti-florigen complex. The three *CmFTL* products all appear to be involved in the regulation of flowering time. However, both the nature of the cross-talk between photoperiod and endogenous sucrose level during floral induction and how the *CmFTLs* function in the regulatory network currently remain unknown. The results from this study suggest that the existence of a sugar-induced *CmFTL* pathway cannot be excluded and might play an important role in the photoperiod- and sucrose-mediated control of flowering time in chrysanthemum. Further investigations to determine the role of *CmFTL* genes in the growth and flowering of chrysanthemums will provide clues for a better understanding of this process.

### Conclusions

The rescue of the delayed flowering phenotype of the *A. thaliana ft-10* mutant by *CmFTL2* confirmed that *CmFTL2* is a florigen. *CmFTL2* was active during the process of floral transition under an SD or an SD+NB regime and much more strongly induced than either *CmFTL1* or *CmFTL3* by sucrose, which might be involved in the flowering of ‘Floral Yuuka’.

### Availability of data and materials

All the data supporting our findings are contained within the manuscript, in text, tables and figures and in the Supplementary Files.
