# Supplementary material for: CmFTL2 is involved in the photoperiod- and sucrose-mediated control of flowering time in chrysanthemum
Source: Hortic Res. 2017 Feb 15;4:17001–. doi: 10.1038/hortres.2017.1 (PMC5314951; doi:10.1038/hortres.2017.1)
Supplement: Supplementary Table S1 [file hortres20171-s1.doc]

| Table S1. Oligonucleotides used in this study. | |  |
| --- | --- | --- |
| Name | Sequence (5' to 3') | Note |
| qCmFTL2-F | ATGTGTTATTCCGGCAATTGGGTCG | qPCR for *CmFTL2* in chrysanthemum |
| qCmFTL2-R | AAATATGCATTTGTAACGTCATGTG | qPCR for *CmFTL2* in chrysanthemum |
| qCmM111F | TCATGCCACATGAGGTGCTTTC | qPCR for *CmM111* |
| qCmM111R | TCGGTAGAGCATTGATGGATCG | qPCR for *CmM111* |
| EF1α-F | TTTTGGTATCTGGTCCTGGAG | qPCR for *EF1α* |
| EF1α-R | CCATTCAAGCGACAGACTCA | qPCR for *EF1α* |
| CmFT2qF | CGTAATAGGGGATGTTCTTGATAGT | qPCR for *CmFTL2* in Arabidopsis |
| CmFT2qR | AGCATCAGGATCAACCATCACTAAT | qPCR for *CmFTL2* in Arabidopsis |
| CmFTL2-F | TTGCGGCCGCTAATGCCGAGGGAAAGGGAT | Cloning |
| CmFTL2-R | TGATGTGCGTGCTTTCAAAATATGC | Cloning |
| qCmFTL3-F | CTATGAGAGCCCAAGGCCATCAATG | qPCR for *CmFTL3* in chrysanthemum |
| qCmFTL3-R | TGATGTTCGTGCTTTCAATATGTAT | qPCR for *CmFTL3* in chrysanthemum |
| qCmFTL1-F | AATCGTGTGCTATGAGAGCC | qPCR for *CmFTL1* in chrysanthemum |
| qCmFTL1-R | GCTTGTAACGTCCTCTTCATGC | qPCR for *CmFTL1* in chrysanthemum |
| ACT2-F | CTTACCTTGAAGTATCCTATTGAGC | qPCR for *Actin2* in Arabidopsis |
| ACT2-R | TGGATGGCGACATACATAGCGGGAG | qPCR for *Actin2* in Arabidopsis |
